# Improved hatchability and efficient protection after *in ovo *vaccination with live-attenuated H7N2 and H9N2 avian influenza viruses

**DOI:** 10.1186/1743-422X-8-31

**Published:** 2011-01-21

**Authors:** Yibin Cai, Haichen Song, Jianqiang Ye, Hongxia Shao, Rangarajan Padmanabhan, Troy C Sutton, Daniel R Perez

**Affiliations:** 1 Department of Veterinary Medicine, University of Maryland, College Park, 8075 Greenmead Drive, College Park, MD 20742-3711, USA; 2 Virginia-Maryland Regional College of Veterinary Medicine, 8075 Greenmead Drive, College Park, MD 20742-3711, USA; 3 Synbiotics Co. 8075 Greenmead Drive, College Park, MD 20742-3711, USA; 4 Department of Animal and Avian Sciences, University of Maryland College Park, 1413 Animal Sciences Center, College Park, MD 20742-2311, USA

## Abstract

Mass *in ovo *vaccination with live attenuated viruses is widely used in the poultry industry to protect against various infectious diseases. The worldwide outbreaks of low pathogenic and highly pathogenic avian influenza highlight the pressing need for the development of similar mass vaccination strategies against avian influenza viruses. We have previously shown that a genetically modified live attenuated avian influenza virus (LAIV) was amenable for *in ovo *vaccination and provided optimal protection against H5 HPAI viruses. However, *in ovo *vaccination against other subtypes resulted in poor hatchability and, therefore, seemed impractical. In this study, we modified the H7 and H9 hemagglutinin (HA) proteins by substituting the amino acids at the cleavage site for those found in the H6 HA subtype. We found that with this modification, a single dose *in ovo *vaccination of 18-day old eggs provided complete protection against homologous challenge with low pathogenic virus in ≥70% of chickens at 2 or 6 weeks post-hatching. Further, inoculation of 19-day old egg embryos with 10^6 ^EID_50 _of LAIVs improved hatchability to ≥90% (equivalent to unvaccinated controls) with similar levels of protection. Our findings indicate that the strategy of modifying the HA cleavage site combined with the LAIV backbone could be used for *in ovo *vaccination against avian influenza. Importantly, with protection conferred as early as 2 weeks post-hatching, with this strategy birds would be protected prior to or at the time of delivery to a farm or commercial operation.

## Introduction

Although depopulation of infected flocks is the method of choice to control the spread of avian Influenza virus (AIV) in poultry, vaccination has become an alternative strategy in order to provide protection to high-risk birds and reduce the possibility of transmission among birds and/or to mammals [1,2]. Thus, in many countries in which avian influenza outbreaks particularly of low pathogenicity have occurred recurrently, selective culling followed by vaccination is used as a measure to control the disease without major economic disruptions. There are only two types of avian influenza vaccines (AIVs) licensed worldwide: inactivated whole AIV vaccine and recombinant fowlpox virus-vectored vaccine expressing the HA gene of AIV. However, both types of vaccines have major limitations: inactivated vaccines cannot elicit strong mucosal and cellular immunity; and previous exposure to fowlpox virus inhibits the host response to the fowl-pox vectored vaccine inhibiting anti-influenza immunity [2-4]. In addition, both strategies are heavily time-consuming, requiring each bird to be vaccinated individually by parenteral inoculation.

With the advent of reverse genetics, LAIVs have emerged as a potential alternative to control avian influenza [5]. Several different strategies have been developed to attenuate influenza viruses based on mutations in one or more of the viral internal or surface genes [6-9]. Several studies have shown that LAIV vaccines protect against influenza viruses of low or high pathogenicity in poultry and mammals. However, field application of these vaccines is difficult due to the inherent segmented nature of the influenza genome and the fear that LAIVs could expand the plethora of influenza viruses through reassortment. Despite recent reports of the potential genomic manipulation of influenza to prevent undesired reassortments, it is unclear how these viruses will behave under more natural conditions; either by providing adequate protection or reverting to wild type-like viruses. Instead, *in ovo *vaccination using LAIV is an attractive alternative to provide fast and effective protection against influenza while avoiding the potential for reassortment (*in ovo *vaccination is unlikely to produce reassortants as other influenza viruses are not present in the egg).

Several strategies have been developed to generate LAIVs for *in ovo *vaccination. A recombinant LAIV was recently developed that provided immunity against HPAI H5N1 influenza and Newcastle Disease Virus (NDV) [7,10]. This recombinant influenza virus expressed the HA of H5 with a deleted polybasic cleavage site, and the ectodomain of the hemagglutinin-neuraminidase (HN) genes NDV instead of NA gene of HPAI H5N1. With this bivalent virus, a single dose *in ovo *vaccination of 18-day-old eggs provided 90% and 80% protection as early as 3 weeks post-hatching, against NDV and HPAI, respectively. A second strategy employed a non-replicating human adenovirus serotype 5 (Ad5)- vectored vaccine that expressed the HA of a LPAI H5N9 virus. Similarly, this vaccine was delivered *in ovo *and conferred protection in chickens after challenge with either HPAI H5N1 (89% HA homology; 68% protection) or HPAI H5N2 (94% HA homology; 100% protection) viruses. Unfortunately, in both these studies, the hatchability efficiency was not addressed in detail [11].

In our previous reports we demonstrated the potential of a genetically modified LAIV with the internal gene backbone of A/guinea fowl/Hong Kong/WF10/99 (H9N2) (WF10*att*) as a vaccine backbone for H5N1 influenza viruses [2]. The WF10*att *backbone carries mutations in the PB1 (K391E, E581G and A661T) and PB2 (N265S) genes. In addition an HA tag was cloned in frame at the C-terminus of PB1, and enhanced the *att *phenotype. This backbone results in virus attenuation *in vitro *while attaining high viral growth properties at the permissive temperatures of 33 and 35°C. We also showed that an H5N1 virus carrying the backbone ΔH5N1WF10*att *was amenable for *in ovo *vaccination and provided optimal protection against H5 HPAI virus. More specifically, a single low (10^4 ^EID_50_) or high (10^6 ^EID_50_) dose of LAIV resulted in greater than 60% protection at 4-week post-hatching and 100% protection at 9 to 12-week post-hatching. Incorporation of a boost regime with either the low or high virus dose at 2-weeks post-hatching increased the protection efficiency to 100% in 4-week old chickens. The hatchability efficiency of the high-dose (10^6 ^EID_50_) *in ovo *vaccination was 85%, compared with 90% in low-dose (10^4 ^EID_50_) and mock groups [2,12].

*In ovo *vaccination with live attenuated viruses is widely used in commercial poultry against various infectious diseases. *In ovo *vaccination was initially introduced into the poultry market to protect against Marek's disease virus (MD) [13,14]. Currently, over 80% of US broilers are immunized *in ovo *with MD vaccine. *In ovo *vaccination is also effective and used commercially to protect poultry from infectious bursal disease virus (IBDV) [15]. Compared with field vaccination, *in ovo *vaccination provides uniform and fast delivery (50,000 egg/h), reduced labor costs, decreased stress to the birds; and most importantly, elicits early immune responses, as soon as 2-week post hatching [16]. From practical and commercial perspectives, *in ovo *vaccination not only has to be effective in providing protection but also has to maintain high hatchability levels (≥90%). In this report, we investigated the effects of changing the H7 and H9 cleavage site to that of the LPAI H6 subtype and the timing of vaccination on levels of protection and hatchability after *in ovo *vaccination with LAIV against H7 and H9 LPAI viruses. Our results indicate that *in ovo *vaccination can result in significant protection against the H7 and H9 virus subtypes while maintaining high hatchability (>90%) when the vaccine is administered in 19-day old chicken embryos.

## Materials and methods

### Viruses, cells and animals

The influenza virus A/Guinea Fowl/Hong Kong/WF10/99 (H9N2) (WF10) was kindly provided by Robert Webster from the repository at St. Jude's Children's Research Hospital, Memphis, Tennessee; influenza virus A/Chicken/Delaware/VIVA/04 (H7N2) (CK/04) was kindly obtained from Dennis Senne at the National Veterinary Laboratory Services, USDA, Ames, Iowa. The viruses were propagated in 10-day-old embryonated specific-pathogen-free chicken eggs at 35°C and stored at -70°C. The viruses were titrated by the Reed and Muench method to determine the 50% egg infectious dose (EID_50_) [17]. 293T human embryonic kidney and Madin-Darby canine kidney (MDCK) cells were maintained as described previously [2]. White leghorn chickens (Charles River Laboratories, MA) and Japanese quail (Murray McMurray Hatchery, Webster, IA) were hatched at 100°F in a circulating air incubator (G.Q.F. Manufacturing co. Savannah, GA) and maintained under BSL2 conditions.

### Generation of recombinant virus by reverse genetics

**Figure 1:**
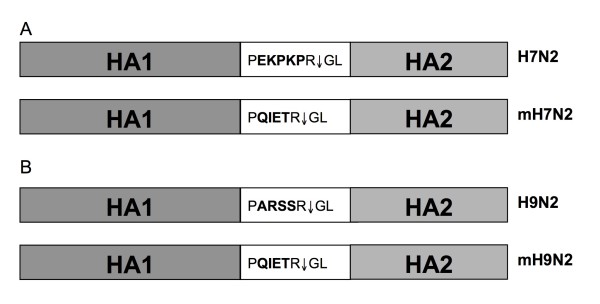
**Strategy of modifying the HA cleavage site**. (A). The substitution of H7N2 (VIVA/04) HA amino acid cleavage site with alternative cleavage site sequences of H6's. (B). The substitution of H9N2 (WF10) HA amino acid cleavage site with alternative cleavage site sequences of H6's.

The 6 internal genes of WF10*att *were described previously and were used to recover viruses carrying the surface genes of Ck/04 or WF10 [2]. The cloning of the Ck/04 surface genes has been previously described [2]. The H7 HA cleavage site, PEKPKPRG, was substituted with an alternative cleavage site sequence, PQIETRG, from the H6 HA subtype using a two-step PCR reaction and the plasmid pDP2002-H7 (Ck/04) as the template (Figure 1A). In brief, two PCR fragments were produced by using primers EcoR I 550-F (5'-CTGTCGAATTCAGATAATTCAGC-3') and H7-H6 CVS-R (5'-GGTCTCCCGCTGTGGAACATTTCTC-3'), and primers H7-H6 CVS-F (5'-CACAGCGGGAGACCAGAGGCCTTTTTG-3') and Pst I 1150-R (5'-GTCAGCTGCAGTTCCCTCCCCTTGT-3'). These two fragments were then used as templates for a new PCR product using primers EcoR I 550-F and Pst I 1150-R. The fragment was digested with EcoR I and Pst I, and cloned into pDP-2002-H7 (VIVA/04), to obtain pDP2002-mH7.

The H9 HA cleavage site, PARSSRG, was substituted with the alternative cleavage site sequence PQIETRG (Figure 1B) using pDPH9WF10 as the template. Two PCR fragments were produced by using primers: Xbal I 285-F (5'-CCTCATTCTAGACACATGCAC-3') and H9-H6 CVS-R (5'-CCAAATAGTCCTCTAGTTTCGATCTGAGGCACGTTC-3'), and primers H9-H6 CVS-F (5'-GAACGTGCCTCAGATCGAAACTAGAGGACTATTTGG-3') and EcoN I 1297-R (5'-CCTCATTCTAGACACATGCAC-3'). These two fragments were then used as templates to generate a new PCR fragment using primers Xbal I 285-F and EcoN I 1297-R. The fragment was digested with Xbal I and EcoN I, and cloned into pDPH9WF10, resulting in the formation of pDP-2002-mH9.

Recombinant viruses were generated using the 8 plasmid system in co-cultured 293T and MDCK cells as described previously [2]. The recombinant viruses (Table 1) were propagated in 10-day-old embryonated eggs, titrated by EID_50_, and stored at -70°C until use. 2mH7N2:6WF10*att *and 2mH9N2:6WF10*att *viruses were sequenced using specific primers, the Big Dye Terminator v3.1 Cycle Sequencing kit (Applied Biosystems, Foster City, CA), and a 3100 Genetic Analyzer (Applied Biosystems, Foster City, CA), according to the manufacturer's instructions. The genetic stability of mutations on HA, PB1 and PB2 were evaluated by serial passage of virus stocks at a 1:10,000 dilution for 10 passages in triplicate samples in 10-day-old embryonated eggs. Viruses obtained after ten passages were sequenced as described above.

**Table 1:** Gene constellations of recombinant viruses used in this study.

Virus	HA	NA	Internal genes (PB1, PB2, PA, NP, M and NS)
2m2H7N2:6WF10*att*	mH7 (VIVA/04)	N2 (VIVA/04)	WF10*att*

2H7N2:6WF10*att*	H7 (VIVA/04)	N2 (VIVA/04)	WF10*att*

2mH9N2:6WF10*att*	mH9 (WF10)	N2 (WF10)	WF10*att*

2H9N2:6WF10*att*	H9 (WF10)	N2 (WF10)	WF10*att*

### Hatchability in embryonated chicken eggs

18 or 19-day-old embryonated specific-pathogen-free chicken eggs were inoculated with either 10^6 ^or 10^7 ^EID_50 _of virus in 0.1 ml inoculum according to the scheme presented in Table 2. Eggs in the mock group were inoculated with 0.1 ml of PBS. The egg inoculation was performed as described previously [2]. Briefly, eggs were candled, and a small hole was made through the air cell with an electric drill. Next, 0.1 ml of virus dilution or PBS was injected into the allantoic cavity using a 21-gauge needle at a depth of 2.5 cm. The percent hatchability was calculated using the total number of inoculated eggs versus the number of 21-day old eggs that hatched in each group. This experiment was performed under BSL-2 conditions according to protocols approved by the Animal Care and Use Committee of the University of Maryland.

**Table 2:** Comparison of the hatchability of new recombinant viruses in embryonated chicken eggs vs. the viruses with wild type HAs and the optimization of the dose and timing for *in-ovo *vaccination.

Vaccine		Dose (EID50)	Embryo age (Day)	% Hatchability (# hatched/total #)
H7N2 (VIVA/04) Vaccine	2mH7N2:6WF10 *att*	10^6^	18	50% (15/30)
				(P = 0.016)
		
		10^6^	19	93% (42/45)
				(P = 0.061)
		
		10^7^	19	80% (24/30)
				(P = 0.066)
	
	2H7N2:6WF10*att*	10^6^	18	30% (9/30)
		
		10^6^	19	43% (13/30)
		
		10^7^	19	37% (11/30)

H9N2 (WF10) Vaccine	2mH9N2:6WF10*att*	10^6^	18	63% (19/30)
				(P = 0.0161)
		
		10^6^	19	90% (27/30)
				(P = 0.260)
		
		10^7^	19	83% (25/30)
				(P = 0.154)
	
	2H9N2:6WF10*att*	10^6^	18	37% (11/30)
		
		10^6^	19	60% (18/30)
		
		10^7^	19	37% (11/30)

PBS (Mock)		0	18	93% (28/30)
		
		0	19	96% (43/45)

* 3 chickens dead at 2-5 days post-hatching.

### Plaque assay in chicken embryonic kidney (CEK) cells and immunostaining

To investigate if the replacement of amino acids at the HA cleavage site affected the temperature sensitive phenotype of the new live-attenuated viruses, plaque assays were performed in CEK cells at 37°C, 39°C, and 41°C. Confluent CEK cell monolayers in six-well plates were infected with 0.5 ml of 10-fold dilutions of virus 2mH7N2:6WF10*att *or 2H7N2:6WF10*att *in M199 medium. The cells were incubated with the virus dilutions for 1 h at 37°C, washed, and overlaid with M199 medium containing 0.9% agar and 0.1 μg/ml TPCK-trypsin. The plates were then incubated at 37°C, 39°C, and 41°C with 5% CO_2_. At 4 days post-inoculation (dpi) the overlay was removed and immunostaining was performed as described previously [2]. In brief, the cells were fixed, permeabilized, and blocked with bovine serum albumin (BSA) in PBS. The cells were then incubated with mouse anti-WF10 monoclonal NP antibody prepared in our laboratory, followed by incubation with peroxidase-conjugated goat anti-mouse IgG (Jackson Immuno Research, West Grove, PA). The presence of viral antigen was revealed by adding several drops of aminoethylcarbazol (BD Biosciences, San Diego, CA). The size and number of plaques at each temperature were compared to determine the temperature sensitive phenotype of the new recombinant virus.

### Viral replication in MDCK cells

Viral replication was studied to examine the temperature sensitive phenotype of the new recombinant viruses in MDCK cells. Confluent monolayers of MDCK cells in 6-well plates were infected with 2m2H7N2:6WF10*att *or 2H7N2:6WF10*att *at a MOI = 0.001 and cultured at 35°C and 39°C, respectively. Supernatant samples were collected at 12, 24, 48, 72, 96 and 120 h post-inoculation, and the viral titer of these samples was determined by TCID_50 _in MDCK cells [2].

### Virus replication and transmission in quail

To evaluate the vaccine's attenuated phenotype *in vivo*, 2mH7N2:6WF10*att *was compared to the recombinant virus 2H7N2:6WF10*att*. Six 4-week-old Japanese quail were inoculated by the ocular, intranasal, and intratracheal routes with 10^6 ^EID_50_/0.5 ml of either 2mH7N2:6WF10*att *or 2H7N2:6WF10*att *vaccine viruses. Two control quail were inoculated with 0.5 ml of PBS. At 1 dpi, 3 naïve quail were introduced into the same isolators, and placed in direct contact with the inoculated quail to assess virus transmission. At 3 dpi, 3 inoculated quail per group were sacrificed, lungs were homogenized and virus titers were determined by EID_50_. For the remaining quail, tracheal and cloacal swabs were collected from both the inoculated and direct contact birds at 1, 3, 5, 7, and 9 dpi. The swab samples were stored in glass vials in 1.0 ml freezing Brain Heart Infusion (BHI) medium (BD, Sparks, MD) and titrated for infectivity in 10-day-old embryonated chicken eggs and MDCK cells. Sera were collected 2 weeks post-infection and HA inhibition tests (HI) were performed to quantify antibodies against HA [18].

### Challenge studies

Chickens that hatched after *in ovo *vaccination were randomly divided into two groups with the same number of individuals. Early protection was assessed in the first group of chickens by challenge at 2-weeks post-hatching. Challenge virus consisted of 5 × 10^5 ^EID_50 _of virus (equal to 500 chicken infectious dose 50 (CID_50_)) and was delivered via intranasal inoculation. Late protection was assessed in the second group of chickens following the strategy described above, but in chickens that were 6 weeks old. Tracheal and cloacal swab samples were collected at 3, 5, and 7 days post-challenge (dpc). Virus shedding was titrated in MDCK cells by TCID_50_. Sera samples were collected at 2-weeks post-hatching pre-challenge, and 2 weeks post-challenge. HI titers were determined as previously described [18]. Animal studies were conducted under BSL-2 conditions, and performed according to protocols approved by the Animal Care and Use Committee of the University of Maryland.

## Results

### Chicken hatchability is impaired after *in ovo *vaccination with H7N2 and H9N2 WF10*att *viruses

Our previous studies showed that *in ovo *vaccination with 10^6 ^EID_50 _of the ΔH5N1:6WF10*att *virus resulted in effective protection against HPAI H5N1 virus [2]. We wanted to determine whether similar levels of protection could be obtained against other HA subtypes following the same strategy. We were particularly interested in the H7 and the H9 subtypes because they have been responsible for recurrent outbreaks, particularly in Eurasia (although in our studies a H7 virus of the North American lineage was used). Thus, 18-day-old egg embryos were inoculated with 10^6 ^EID_50 _of either 2H7N2:6WF10*att *or 2H9N2:6WF10*att *vaccine viruses (Tables 1 and 2). Unfortunately, the hatchability of vaccinated eggs was poor, 30% and 37% in eggs vaccinated with 2H7N2:6WF10*att *and 2H9N2:6WF10*att*, respectively (Table 2) compared to 85% in eggs vaccinated with the 2ΔH5N1:6WF10*att *virus (not shown and [2]).

### Chicken hatchability after modification of the HA cleavage site in H7N2 and H9N2 WF10*att *viruses

**Figure 2:**
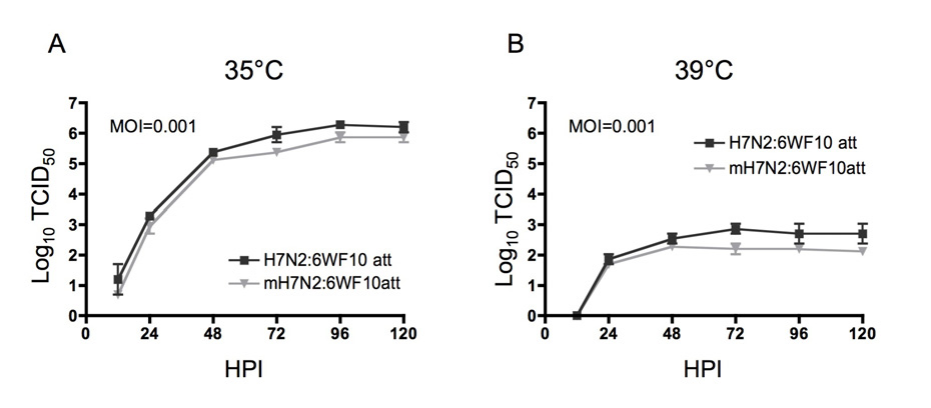
**Viral replication kinetics of the live-attenuated viruses in MDCK cells at (A) 35°C and (B) 39°C using MOI of 0.001**. Viral titers at different time points were determined by TCID_50_.

**Figure 3:**
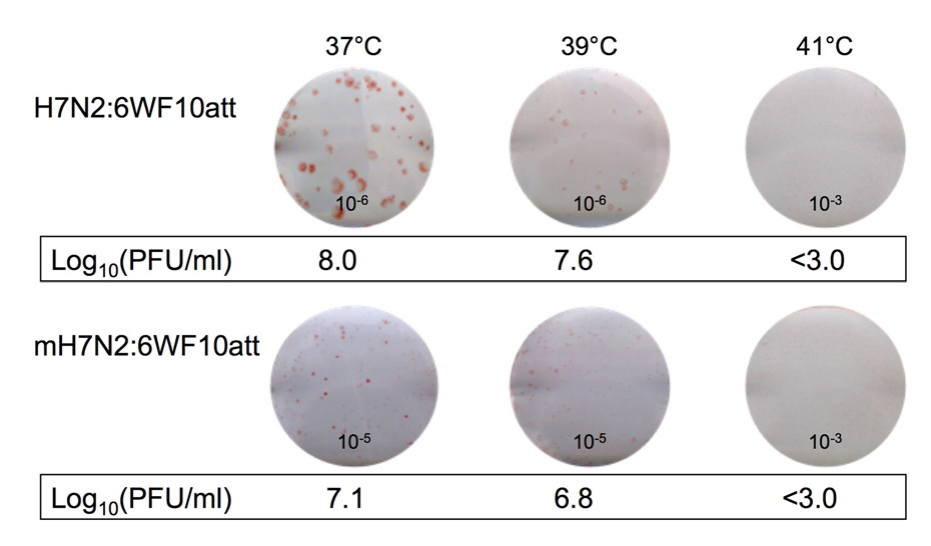
**Plaque morphologies of the live-attenuated viruses in CEK cell at different temperatures**. Confluent CEK cells in six-well plates were infected with 2mH7N2:6WF10*att *or 2H7N2:6WF10*att*. The numbers 10^-6^, 10^-5^, and 10^-3 ^on the plaque pictures indicate the virus dilution used to infect cells at the indicated temperature. The cells incubated at 37°C, 39°C, or 41°C, respectively, for 4 days post infection and then fixed and the viral antigen was visualized by immunostaining as described in Materials and Methods. The plaques sizes were observed and the plaque numbers were counted and calculated as the log_10 _PFU/ml, as indicated below the individual plaque picture. A titer of <3.0 log_10 _PFU/ml indicates that no virus was detected at 10^-3 ^dilution.

The 2ΔH5N1:6WF10*att *virus carries the H5 HA protein from A/Vietnam/1203/04 (H5N1) but its polybasic cleavage site, characteristic of HPAI viruses, has been replaced with that from the LPAI H6 HA virus subtype, as described in previous reports [19]. In order to determine if incorporation of the H6 HA cleavage site in the H7 and H9 subtypes would result in more attenuated vaccine viruses and improved hatchability, we generated the recombinant viruses 2mH7N2:6WF10*att *and 2mH9N2:6WF10*att*. Modifications at the cleavage site in these viruses did not have major effects on the *in vitro *properties of these viruses. Both recombinant viruses reached titers of 10^6 ^TCID_50_/ml at 120 h post-infection in MDCK cells inoculated at an MOI = 0.001 and cultured at 35°C (Figure 2 and data not shown). In contrast, viral replication at 39°C was severely restricted, with viral titers reduced more than 1000-fold relative to those at 35°C (Figure 2 and data not shown). This indicates that modifications in the HA cleavage site did not change the temperature sensitive phenotype of these viruses in MDCK cells. Likewise, plaque assays, performed using CEK cells (Figure 3), showed that 2mH7N2:6WF10*att *formed significantly smaller plaques than 2H7N2:6WF10*att *at 37° and 39°C. As expected, these viruses were highly restricted at 41°C (yields of <10^3 ^PFU/ml) consistent with their *att *phenotype. Interestingly, the lower virus titers and smaller plaque sizes of 2mH7N2:6WF10*att *compared to 2H7N2:6WF10*att *indicate an additive effect on attenuation provided by the modified HA cleavage site. Similar results were obtained when we compared the 2mH9N2:6WF10*att *to 2H9N2:6WF10*att *(not shown). However, despite the additional attenuation, only a slight improvement in hatchability (50% and 63%) was observed when 18-day-old egg embryos were inoculated with 10^6 ^EID_50 _of the 2mH7N2:6WF10*att *and 2mH9N2:6WF10*att *vaccine viruses, respectively (Table 2).

### Chicken hatchability is improved when *in ovo *vaccination is performed on 19-day old chicken embryos

The previous hatchability results suggested that additional mutations in the virus genome were required or that the conditions under which the vaccine was delivered needed to be changed to improve hatchability. Certainly, additional mutations in the viral genome could be introduced, however, they might also affect immunogenicity.

Thus, we chose to deliver the vaccine to 19-day old chicken embryos and compare hatchability to vaccination of 18-day old chicken embryos. *In ovo *vaccination of 19-day old chicken embryos was performed with either 10^6 ^or 10^7 ^EID_50 _to explore hatchability efficiency with two different virus concentrations. Interestingly, hatchability was greatly improved in 19-day-old vaccinated embryos. Hatchability reached 93% and 90% in the 2mH7N2:6WF10*att *and 2mH9N2:6WF10*att *groups, respectively, when eggs were vaccinated with 10^6 ^EID_50 _(Table 2). As shown in Table 2, an increase in virus delivery dose to 10^7 ^EID_50 _was detrimental for hatching. These results suggest that *in ovo *vaccination in 19-day old chicken embryos may be a suitable strategy to generate an anti-influenza response in chickens.

### Modification of the HA cleavage site reduces replication of 2mH7N2:6WF10*att *virus in quail

We have previously shown that quail are more susceptible than chickens to avian influenza viruses. Thus quail represent a better host to test whether modifications in our vaccine viruses would have any effect on replication and transmissibility. To investigate if modification of the HA cleavage site altered the degree of attenuation and transmissibility in quail, 2 groups of quail (n = 6) were inoculated with either the 2mH7N2:6WF10*att *virus or the 2H7N2: 6WF10*att *virus. At 24 h after infection, 3-naïve quail/group were brought in direct contact with inoculated quail to monitor for transmission (Table 3). At 3 dpi, 3 inoculated quail from each group were sacrificed to determine virus load in the lungs. No virus was detected in the lungs of inoculated quail regardless of the virus used. This finding is consistent with our previous study showing that the WF10*att *backbone prevents the virus from replicating in the lower respiratory tract (not shown and [2,12]). In addition, no virus was detected in cloacal swabs for any of the quail in the study (not shown). In contrast, tracheal swabs showed the presence of virus in the 2H7N2:6WF10*att *group, with peak virus titers of 10^2.9 ^(at 1 dpi) and 10^1.6 ^TCID_50_/ml (at 3 dpi) in the inoculated and direct contact quail, respectively. Inoculated quail remained positive until 5 dpi but were negative by 7 dpi. Only 2 out of the 3 direct contact quail showed trace amounts of 2H7N2:6WF10*att *and were negative by 9 dpi. With respect to the 2mH7N2: 6WF10*att *inoculated group, only trace amounts of virus were observed, and just 1 of 3 quail remained positive by 7 dpi and it became negative by 9 dpi. Direct contacts in the 2mH7N2: 6WF10*att *virus group were negative except for trace amounts of virus on a single day, 7 dpi, in 2 of the 3 quail. The levels of virus replication in the different groups corresponded with the levels of seroconversion observed. Thus, inoculated quail in the 2H7N2:6WF10*att *group had the highest neutralizing antibody response, followed by inoculated quail in the 2mH7N2: 6WF10*att *group, whereas the direct contacts in the 2H7N2:6WF10*att *showed low, but significant seroconversion. Also consistent with the transient presence of the 2mH7N2: 6WF10*att *virus in the direct contact group, very low seroconversion was observed. These studies suggest that alterations in the HA cleavage site have an effect on replication *in vivo *further attenuating these viruses and limiting the ability to replicate after transmission (Table 3). We did not perform similar studies in quail with the H9N2 vaccine viruses. However, we must note that similar studies in white leghorn chickens did not result in detectable transmission, when the viruses carry the *att *backbone in the context of H7N2 or H9N2 surface genes (not shown).

**Table 3:** Replication and transmission study of recombinant virus 2H7N2:6WF10*att *and 2mH7N2:6WF10*att *in quail

Virus	Group	**# of positive tracheal swab/total # post-inoculation (log**_**10**_**TCID**_**50**_**/ml ± SD) at peak viral shedding**					# of seroconverted/total # (Average HI titer at 14 dpi)
			
		1 dpi	3 dpi	5dpi	7 dpi	9 dpi	
2H7N2:6WF10*att*	Inoculated	6/6 (2.9 ± 0.4)	6/6*	3/3	0/3	0/3	3/3 (133)
	
	Contact	NA	3/3 (1.6 ± 1.4)	3/3	2/3	0/3	3/3 (47)

2mH7N2:6WF10*att*	Inoculated	6/6 (<0.7)	6/6*	1/3	1/3	0/3	3/3 (87)
	
	Contact	NA	0/3	0/3	2/3 (<0.7)	0/3	2/3 (10)

* 3 quail from each inoculated group were sacrificed at 3 dpi to determine virus load in the lungs.

### Stability of new recombinant viruses

The genetic stability of the mutations on HA, PB1, and PB2, was verified by serial passage of the 2mH7N2:6WF10*att *and 2mH9N2:6WF10*att *viruses in 10-day-old embryonated eggs. Amino acids 391E, 581G, 661T and the HA tag on PB1, and 265S on PB2 remained unchanged after serial propagation in eggs. More importantly, the amino acids at the HA cleavage site remained unchanged and corresponded to the H6 HA cleavage sequence (PQIETRG).

### Single dose *in ovo *vaccination provides protection in chickens from homologous challenge with H7 and H9 LPAI viruses at 2 and 6 weeks post-hatching

To further evaluate whether *in ovo *immunization would result in protection against H7 or H9 viruses, vaccinated chickens were divided into two groups, and subsequently challenged with homologous virus at either 2 or 6 weeks post-hatching (Tables 4 and 5).

**Table 4:** Single-dose 2mH7N2:6WF10*att in-ovo *vaccination study in chickens challenged with low-pathogenic H7N2 (Ck/04) at 2 and 6 weeks post-hatching

Vaccine dose (EID_50_)/embryo age (days)	# positive HI/total # pre-challenge (HI titer)	Age (in weeks) at time of challenge	**# Shedding virus/total # in swabs (log**_**10**_**TCID**_**50**_**/ml ± SD)**						# positive HI/total # at 14 dpi
			Tracheal			Cloacal			
				
			3 dpc	5 dpc	7 dpc	3 dpc	5 dpc	7 dpc	
0 (Mock)	0/8	2	8/8 (3.4 ± 0.8)	8/8 (2.9 ± 0.6)	0/8	2/8 (3.7)	5/8 (3.4 ± 0.2)	5/8 (3.2 ± 0.5)	8/8 (170)
10^6^, 18	1/4 (3)	2	3/4 (3.3 ± 1.0)	3/4 (2.9 ± 0.9)	0/4	2/4 (4.5 ± 0.7)	3/4 (3.7 ± 1.0)	3/4 (3.7 ± 0.7)	4/4 (320)
**10**^**6**^**, 19**	**6/8 (13)**	**2**	**2/8 (3.5 ± 0.7)**	**1/8 (2.3)**	**0/8**	**0/8**	**0/8**	**1/8 (2.0)**	**8/8 (240)**
10^7^, 19	3/5 (5)	2	1/5 (2.7)	0/5	0/5	0/5	0/5	1/5 (2.3)	5/5 (272)

0 (Mock)	0/7	6	7/7 (3.5 ± 0.7)	7/7 (3.4 ± 0.7)	0/7	3/7 (3.9 ± 0.5)	5/7 (3.7 ± 1.0)	5/7 (3.3 ± 0.8)	7/7 (525)
10^6^, 18	2/4 (50)	6	2/4 (4.1 ± 0.6)	2/4 (3.9 ± 0.6)	0/4	1/4 (3.5)	2/4 (4.3 ± 0.4)	2/4 (3.6 ± 0.1)	4/4 (360)
**10**^**6**^**, 19**	**5/7 (51)**	**6**	**2/7 (3.4 ± 0.2)**	**0/7**	**0/7**	**1/7 (3.7)**	**1/7 (3.5)**	**1/7 (3.3)**	**7/7 (525)**
10^7^, 19	4/5 (64)	6	1/5 (3.7)	1/5 (3.7)	0/5	0/5	0/5	0/5	5/5 (640)

Pre-challenge sera collected at 2 weeks post-hatching showed limited seroconversion in chickens that received the 2mH7N2:6WF10*att *(Table 4), both in terms of the number of seropositive chickens as well as the level of HI responses. However, sera collected at 6 weeks post-hatching showed increased numbers of seropositive chickens and increased HI titers (Table 4). Relative to 2mH7N2:6WF10*att*, improved and more consistent antibody responses were obtained in chickens that were vaccinated with 2mH9N2:6WF10*att *(Table 5). In terms of protection, significant protection was observed in chickens challenged with 500 CID_50 _of Ck/04 (H7N2) at 2 or 6 weeks post-hatching but only in the 19-day old embryo vaccinated groups. Tracheal virus shedding was detected in only 2 out 8 and 1 out of 5 chickens in the 19-day old embryo groups that received 10^6 ^or 10^7 ^EID_50_, respectively, of 2mH7N2:6WF10*att*. There was also a sharp decrease in cloacal virus shedding in these groups, with just 1 out 8 (10^6 ^EID_50 _group) and 1 out 5 (10^7 ^EID_50 _group) virus positive chickens and only at 7 dpc (Table 4). In contrast, in the 18-day old embryo vaccinated group only 1 out 4 and 2 out 4, at 2 and 6 weeks post-hatching, respectively, showed protection and no detectable virus replication. Similar protective responses were observed in the WF10(H9N2) challenged chickens. Chickens in the 19-day old embryo vaccinated groups showing the best protection, and those in the 18-day old embryo vaccinated groups showed the decreased protection (Table 5). Significant seroconversion in all the groups at 14 dpc indicated that lack of virus shedding in protected chickens was not due to a failure in our challenge approach. Considering the 10^6 ^EID_50 _vaccine dose in the 19-day old embryo vaccinated groups for both *att *vaccines, there was between 70 and 80% protection efficiency in chickens challenge at 2 or 6 weeks post-hatching, respectively. Slightly better protection efficiency (82%) was observed in the 10^7 ^EID_50 _vaccine dose groups; however, it was achieved at the expense of lower hatchability rates (~91% for the 10^6 ^EID_50 _versus ~80% for the 10^7 ^EID_50 _groups). In contrast, an average of only 55% protection efficiency was observed in the groups vaccinated with a dose 10^6 ^EID_50 _in 18-day old embryos.

**Table 5:** Single-dose 2mH9N2:6WF10*att in-ovo *vaccination study in chickens challenged with low-pathogenic H9N2 (WF10) at 2 and 6 weeks post-hatching

**Vaccine dose (EID**_**50**_)**/embryo age (days)**	# positive HI/total # before challenge	Age (in weeks) at time of challenge	**# Shedding virus/total # in swabs (log**_**10**_**TCID**_**50**_**/ml ± SD)**			
			
			Tracheal			# positive HI/total # at 14 dpi
				
			3 dpc	5 dpc	7 dpc	
0 (Mock)	0/7	2	7/7 (2.7 ± 0.6)	7/7 (2.5 ± 0.3)	0/7	7/7 (217)
10^6^, 18	3/5 (14)	2	2/5 (2.2 ± 0.2)	2/5 (2.3 ± 0.4)	0/5	5/5 (192)
**10**^**6**^**, 19**	**6/7 (32)**	**2**	**1/7 (2.5)**	**0/7**	**0/7**	**7/7 (286)**
10^7^, 19	3/4 (32)	2	1/4 (2.3)	0/4	0/4	4/4 (260)

0 (Mock)	0/7	6	7/7 (2.5 ± 0.3)	7/7 (2.5 ± 0.2)	0/7	7/7 (320)
10^6^, 18	2/5 (30)	6	3/5 (2.6 ± 0.7)	3/5 (2.2 ± 0.9)	0/5	5/5 (224)
**10**^**6**^**, 19**	**5/7 (71)**	**6**	**2/7 (2.4 ± 0.5)**	**0/7**	**0/7**	**7/7 (446)**
10^7^, 19	3/3 (67)	6	0/3	0/3	0/3	3/3 (227)

## Discussion

The HA is perhaps the most important protein in influenza viruses, as it is a critical determinant of host range and virulence [20,21]. The HA protein, encoded in segment 4, is expressed on the virus surface as homotrimers. It is initially produced as a precursor, HA0, that requires post-translational modifications, including cleavage and glycosylation in order to become fully active [22]. Cleavage of the HA0 precursor leads to two subunits, HA1 - N-proximal - and HA2 - C-proximal -, which are maintained covalently linked via disulfide bonds. Trypsin-like host proteases found in the lumen of the respiratory and intestinal tracts are involved in the cleavage of the HA of low pathogenic avian influenza viruses - LPAIV - (and mammalian influenza viruses) [22]. Intracellular furin-like proteases have been implicated in the cleavage of the HA of highly pathogenic avian influenza viruses - HPAIV [22]. The number of basic amino acid residues preceding the cleavage site determines recognition by either trypsin-like or furin-like proteases, with a string of basic amino acids allowing the latter to cause intracellular maturation of the HA at the level of the endoplasmic reticulum [23]. Furin-like protease cleavage produces mature virions that can spread cell to cell without having to reach the lumen of the respiratory or intestinal tracts. This permits the development of a fatal systemic infection, hence the so-called highly pathogenic influenza. Therefore, the cleavability of HA is one of the critical factors for viral tissue tropism and pathogenicity [24,25]. In this study, we modified the cleavage site of the influenza virus H7 and H9 HA protein genes to encode sequences corresponding to the H6 HA cleavage site (mH7 and mH9) in order to improve hatchability after *in ovo *vaccination. It has been previously shown that the H6 HA cleavage site can transform a HPAIV of the H5N1 subtype into a LPAIV [19]. We have previously shown that a LPAI H5N1 virus carrying *att *mutations is amenable for *in ovo *vaccination resulting in ≥60% protection while maintaining at least 85% hatchability [2]. In this study we sought to examine whether the mH7 and mH9 *att *viruses viruses showed similar replication yields as unmodified H7 and H9 *att *viruses, and if these modified viruses were more amenable for *in ovo *vaccination without decreased immunogenicity. Growth kinetic studies in tissue culture cells showed similar yields for the mH7 compared to the unmodified H7 viruses (Figure 2) and similar results were obtained comparing the mH9 with the unmodified H9 pairs (not shown). As the safe "window" for *in ovo *vaccination of chicken embryos is between day 17 at 12-14 hours to day 19 at 2-4 hours [26], we chose days 18 and 19 for vaccination to test the effects on hatchability of the *att *vaccines. Hatchability studies clearly demonstrated that the mH7 and mH9 *att *viruses allowed for hatchability (90-93%, 19-day old embryos) similar to the PBS inoculated controls (93-96%), which were much higher than those obtained with the unmodified H7 or H9 *att *viruses (43-60%, 19-day old embryos). We found that the combination of the modified HA cleavage site, vaccine dose, and time of vaccine delivery, had a significant impact on hatchability rates. Thus, 18-day old chicken embryos vaccinated with the mH7 or the mH9 *att *viruses showed improved hatchability rates compared to the unmodified HA *att *counterparts, but they were significantly lower than the rates obtained after vaccinating 19-day old embryos (Table 2). Likewise, increasing the dose to 10^7 ^EID_50 _of either mH7 or mH9 *att *viruses resulted in 10% hatchability loss compared to the same age embryos inoculated with 10^6 ^EID_50 _of the same viruses.

We speculate that the introduction of the alternative H6 HA cleavage site in the mH7 and mH9 *att *viruses (and perhaps in the ΔH5 *att *virus) leads to reduced HA cleavage efficiency and, thus, these viruses exhibit growth restrictions at higher temperatures *in vitro *(Figure 3) and *in vivo *in 18-19-day old chicken embryos (Table 2). However, these viruses showed no defects in terms of virus yield at the permissive temperatures of 33 and 35°C in tissue culture (Figure 2) or in 10-day old chicken embryos. These characteristics are important because efficient immunogenicity was maintained without sacrificing virus yield. In fact, 2mH7N2:6WF10*att *and 2mH9N2:6WF10*att *viruses can easily achieve titers on the order of 10^8 ^EID_50_/ml when grown in 10-day old embryonated chicken eggs (data not show), thus making them ideal for mass production.

In ovo vaccination is an attractive approach for vaccination of chickens, particularly broilers [26,27]. It helps to 'close the window' of susceptibility between vaccination and early exposure to infectious agents compared with post-hatch vaccination [27]. Because chickens already develop certain immunologic functions before hatching, *in ovo *vaccination stimulates both the innate and adaptive immune responses. Thus, *in ovo *vaccinated chicks develop an appreciable degree of protection by the time of hatching [27]. This indeed appears to be the case since in our approach chickens showed significant protection (≥ 70%) when challenged as early as 2 weeks post-hatching. It is tempting to speculate that under industrial settings higher protection efficiencies could be obtained since automated systems would result in more accurate, controlled and efficient administration of the vaccine compared to our manual approach. In addition, because the mH7 and mH9 *att *viruses are more attenuated *in vivo *than the unmodified *att *counterparts, we further speculate that these HA genes are not likely to outcompete wild type influenza viruses through reassortment, and thus, should be safe to use in the field. The unprecedented spread of low pathogenic H7 and H9 influenza viruses in commercial settings, calls for the implementation of alternative prevention and control strategies. Our report provides for a viable alternative to the classical vaccination approaches against avian influenza.

## Competing interests

The authors declare that they have no competing interests.

## Authors' contributions

YC designed and performed reverse genetics virus rescue and in ovo vaccination studies and wrote the manuscript. HS perform molecular cloning, animal studies and co-wrote the manuscript. JY, HS, and RP designed and performed animal studies. TCS edited and proofread the manuscript. DRP was responsible for the overall study design, wrote, edited and proofread the manuscript. All authors read and approved the final manuscript.
